# Tolerance of ambiguity and psychological wellbeing in newly qualified doctors: An analysis over multiple time points

**DOI:** 10.1111/medu.15743

**Published:** 2025-06-17

**Authors:** Jason Hancock, Obioha C. Ukoumunne, Bryan Burford, Gillian Vance, Thomas Gale, Karen Mattick

**Affiliations:** ^1^ Health Professionals Education and Wellbeing Research Group, Department of Health and Community Sciences, Faculty of Health and Life Sciences University of Exeter UK; ^2^ Devon Partnership NHS Trust UK; ^3^ NIHR Applied Research Collaboration South West Peninsula (PenARC), Department of Health and Community Sciences, Faculty of Health and Life Sciences University of Exeter UK; ^4^ Medical Education, School of Medicine Newcastle University UK; ^5^ Medical Education, Collaboration for the Advancement of Medical Education Research, Peninsula Medical School University of Plymouth UK

## Abstract

**Introduction:**

There is evidence of an association between tolerance of ambiguity and psychological wellbeing in doctors, but this relationship is not well understood. We explored this relationship, and the individual or workplace factors moderating it, in a population of newly qualified doctors.

**Methods:**

We examined the experiences of newly qualified doctors in the UK as they started a novel interim role (Time 1) and later moved into foundation year 1 roles (Times 2 and 3) during the COVID‐19 pandemic. Doctors completed the Tolerance of Ambiguity of Medical Students and Doctors scale (TAMSAD Range: 0–100), the Perceived Stress Scale (PSS: 0–40), the Hospital Anxiety and Depression Scale (HADS: 0–21) and the Copenhagen Burnout Inventory (CBI: 0–100), over four months. Cross‐sectional and longitudinal relationships between tolerance of ambiguity (TAMSAD) and wellbeing outcomes (PSS, HADS, CBI) were examined and potential moderators (age, gender, recent change in working environment) were explored.

**Results:**

A total of 451 participants completed the survey at Time 1, 214 at Time 2, 172 at Time 3. Higher tolerance of ambiguity was associated with lower levels of stress (regression coefficient: ‐0.09, R2 = 1.6%, p = 0.008), anxiety (−0.06, R2 = 1.6%, p = 0.009), depression (−0.03, R2 = 1.1%, p = 0.03) and workplace burnout (−0.40, R2 = 3.9%, p < 0.001) at Time 1. It was associated with lower levels of anxiety (−0.08, R2 = 2.4%, p = 0.03) at Time 2 and stress (−0.16, R2 = 3.4%, p = 0.02) at Time 3. Individual factors (being over 25 years, being female) and workplace factors (not moving location) seemed to strengthen the relationship between tolerance of ambiguity and psychological wellbeing.

**Conclusion:**

There appears to be a longitudinal relationship between lower tolerance of ambiguity and reduced psychological wellbeing in early career doctors within the UK. This study emphasises the importance of supporting all graduating doctors to navigate clinical ambiguity however further research is needed outside of the context of COVID‐19.

## Abstract 2

Improved tolerance of ambigutiy is known to support improved wellbeing in newly qualified doctors. This study details the role training environment plays in that relationship.

**Abbreviations**
CBI: Copenhagen Burnout Inventory
F1: foundation year 1
FiY1: interim foundation year 1
GMC: General Medical Council
HADS: Hospital Anxiety and Depression Scale
MSC: Medical Schools Council
PSS: Perceived Stress Scale
TAMSAD: tolerance of ambiguity of medical students and doctors scale
ToA: Tolerance of Ambiguity
UKFPO: UK Foundation Programme Office

## 1 INTRODUCTION

Reduced psychological wellbeing of doctors is a huge problem globally.^1^ At the end of undergraduate training, levels of psychological distress amongst medical students are higher than in the age‐matched general population.^2^ In 2020, 40% of UK doctors reported feeling unwell due to work‐related stress^3^ and, in 2022, 22% of doctors reported taking leave due to stress.^4^ Even prior to the COVID‐19 pandemic this represented a significant threat to the medical workforce and the delivery of high‐quality patient care.^5^ Although the prevalence of reduced wellbeing (stress, burnout and common mental health disorders) in doctors is well described, less is known about what might increase the risk of doctors developing these problems.^2^, ^6^ Although there may be a complex and bidirectional relationship between how doctors respond to clinical ambiguity and their own wellbeing, one possibility is that the way doctors respond to ambiguity can influence their likelihood of developing stress, burnout or common mental health conditions.^7^, ^8^

Clinicians frequently encounter ambiguity *(“lack of reliable, credible or adequate information”)* ^9^ in all aspects of their clinical work, which can lead to a perception of uncertainty *(“conscious awareness of ignorance about particular aspects of the world*”).^9^ Hillen and colleagues^9^ propose that an individual's uncertainty tolerance will determine their cognitive, behavioural and emotional responses to uncertainty and that these responses will be moderated by stimulus characteristics, individual characteristics, situational characteristics, cultural and social factors.

One systematic review demonstrated an association between reduced Tolerance of Ambiguity (ToA) and reduced wellbeing in doctors.^7^ However many of the included studies had poor methodological quality,^10^, ^11^ lacked a validity argument for the measurement scales used, and only one study examined this relationship longitudinally.^12^ Its authors propose a conceptual model which outlines the relationship between the stimulus of ambiguity, the individual perception of uncertainty and the subsequent emotional component of uncertainty tolerance which it terms psychological wellbeing (stress, burnout and mental health conditions)^7^ and identified potential moderators to this relationship including personal resilience.^12^, ^13^, ^14^ Although other studies have identified moderators to this relationship such as age,^15^, ^16^ experiences, personal characteristics^17^ and self‐confidence,^18^, ^19^ there may be further individual characteristics that moderate this relationship. For example, there is conflicting evidence regarding the impact of gender on ToA, with some studies demonstrating higher ToA in female medical students,^15^, ^16^ and others suggesting higher ToA in males.^20^, ^21^

There is growing evidence that moderators external to the individual may influence the related construct of uncertainty tolerance. Stephens and colleagues have shown that inclusivity of the working or learning environment, and healthcare professional cultures can influence uncertainty tolerance,^17^ and that anatomy education learning environments can moderate medical students' response to uncertainty.^22^ Ilgen and colleagues suggest that when developing strategies to support trainee doctors to recognise, appraise and manage difficult moments with uncertainty in clinical practice, we should more explicitly consider both the internal and environmental cues that will influence the way a trainee experiences uncertainty.^23^ In a study of ToA in medical students and early postgraduate doctors, levels of ToA were lower in year groups that followed a significant transition, such as the first year of postgraduate training,^24^ although the study was insufficiently powered to draw firm conclusions. This finding mirrors that of a study, which suggests that first‐year postgraduate doctors in the UK find the transition to independent practice stressful and that managing uncertainty in the clinical environment can contribute towards this.^25^

In order to promote wellbeing of doctors, it is important to extend our understanding of the relationship between ToA and wellbeing by utilising valid measurement scales for these constructs, collecting longitudinal data and exploring the strength and potential moderators of this relationship (age, gender, factors related to their working environment and points of transition). Doing so would support researchers to refine existing conceptual models, and support educators to design evidence‐based clinical education interventions to improve wellbeing of clinicians, either through focusing efforts on enhancing ToA or on reducing the impact of reduced ToA on reduced wellbeing. Depending on the moderators identified, this could focus on support for the individual or modifications to the educational or working environments.

### 1.1 Study hypotheses

We use the conceptual model proposed by Hancock and Mattick as our starting point.^7^ We hypothesise that having a higher ToA is likely to be associated with better current wellbeing (Hypothesis 1), and increased likelihood of better wellbeing in the future (Hypothesis 2). We also hypothesise that individual factors (age and gender, Hypothesis 3) and workplace factors (recent changes in working environments, Hypothesis 4) will moderate the relationship between ToA and wellbeing. More specifically that being younger, male and having a recent change in working environment will strengthen the relationship between reduced ToA and wellbeing.^15^, ^16^, ^24^, ^25^

## 2 METHODS

The aim of this study is to explore the cross‐sectional and longitudinal relationship between ToA and wellbeing (stress, burnout and common mental health disorders) using a sample of newly qualified doctors, and the individual or workplace factors that moderate any relationship. The research team approached this study from a post‐positivist paradigm perspective.

### 2.1 Participants and context

In May to July 2020, due to the COVID‐19 pandemic, final‐year medical students in some UK medical schools were able to graduate early, gain provisional registration with the General Medical Council (GMC), and start work in a novel role known as Foundation Interim Year 1 posts (FiY1). Doctors in these roles worked across a range of clinical environments. The composition of the work was comparable to that of traditional postgraduate Foundation year 1 (F1) roles, albeit in the context of the COVID‐19 pandemic.^26^ It was possible for doctors to express preferences for the location of both their FiY1 and F1 roles, and change their working location between medical school and FiY1 and between FiY1 and F1.

### 2.2 Study design and data collection

**FIGURE 1:**
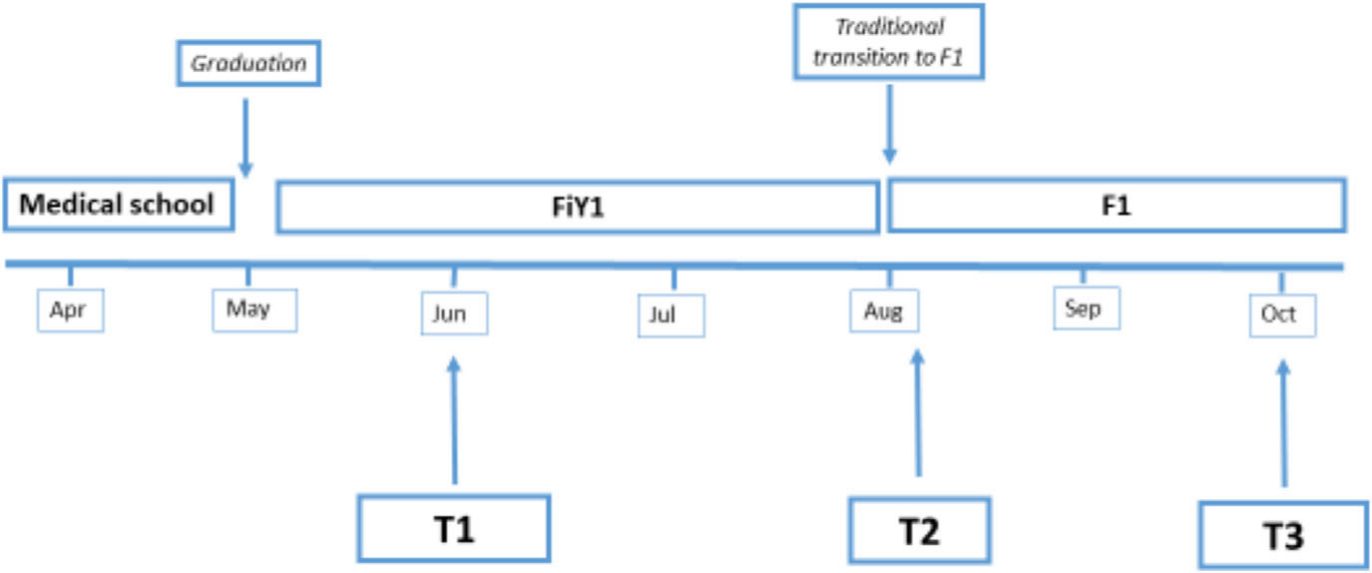
Recruitment timeline 2020: *FiY1 (foundation interim year 1), F1 (foundation year 1), T1 (Timepoint 1 June: novel FiY1 role), T2 (Timepoint 2 August: entry to F1), T3 (Timepoint 3 October: established F1)*. [Color figure can be viewed at wileyonlinelibrary.com]

This was part of a large national mixed methods research project examining the FiY1 role in which quantitative data were collected to explore participants ToA and their wellbeing.^26^, ^27^ Participants signed up to the study in early May 2020 following an email to all UK final year medical students. Timepoint 1 (T1) questionnaire data were collected in June 2020. Timepoint 2 (T2) data were collected in August and Timepoint 3 (T3) data in October (Figure 1). Questionnaires were piloted with four FiY1 doctors and comprised a battery of scales and items relevant to work and wellbeing, including the Tolerance of Ambiguity of Medical Students and Doctors scale (TAMSAD),^24^ the Perceived Stress Scale (PSS),^28^ the Hospital Anxiety and Depression Scale (HADS)^29^ and the work and personal burnout subscales of the Copenhagen Burnout Inventory (CBI).^30^ Demographic details were collected including gender, age, ethnic group and resident UK nation.

The 29‐item TAMSAD scale^24^ contains clinically contextualised statements with participants indicating their response on a five‐point Likert scale (1 to 5) ranging from “strongly disagree” to “strongly agree”. Higher scores indicate higher levels of ToA. A strong validity argument has been made for the use of this scale in the early career doctor population^24^ (Supplementary Table 1) and it has been used in multiple studies.^31^, ^32^, ^33^, ^34^, ^35^, ^36^

The authors selected measures of wellbeing following a literature review of scale validity in this population and discussions with key stakeholders. A strong argument has been made for the use of the HADS anxiety and depression scale in UK‐based medical students,^37^ the CBI scale has been widely utilised for healthcare employees including medical students and doctors,^38^ and the PSS is an internationally accepted and widely used tool in healthcare professionals.^39^ Further details of the validity arguments for the use of these scales in this population and the approaches adopted for scoring are provided (Supplementary Tables 1 and 2).

### 2.3 Data analysis

Summary characteristics of participants enrolling in the study, along with the proportion completing their FiY1 job in the same region as their medical school, and the proportion staying in the same region between FiY1 and F1 were reported. For the purposes of further analysis, all participants who completed a full questionnaire were included at T1, only participants who had previously completed a questionnaire at T1 were included at T2 and only participants who had completed a questionnaire at T1 and T2 were included at T3. Linear regression models were fitted to examine the relationship between ToA (predictor variable: TAMSAD) and wellbeing (stress: PSS, anxiety: HADS, depression: HADS, and burnout: CBI). Cross‐sectional analyses were undertaken to examine the relationship between ToA and wellbeing outcomes at study T1, as well as longitudinal analyses where ToA at T1 was used as a predictor of wellbeing at each of T2 and T3. Crude (unadjusted) linear regression analyses and analyses that were adjusted for potential confounders (age and gender) were undertaken. Estimated regression coefficients are reported with confidence intervals and p values. In exploratory analyses, tests of interaction were undertaken to examine whether age, gender and change in working environment (between medical school and FiY1 or between FiY1 and F1) are moderators of the relationships between ToA and wellbeing. We conducted a separate analysis for each potential moderator for each wellbeing outcome at each time point T1, T2, T3. The p‐value for the interaction between the potential moderator and ToA was reported and used to quantify evidence for moderation. The 5% level was used as the p‐value threshold on the interaction term for evidence of moderation. Where there was evidence of moderation, we reported the regression coefficient and 95% confidence interval for the relationship between ToA and the wellbeing outcome, separately for each subgroup on the potential moderator; separate p‐values were not reported for each subgroup. Given the exploratory nature of the moderator analyses and the number of tests undertaken, evidence of moderation should be treated as provisional, requiring replication in future studies in order to have credence.

The main reported regression results are based on complete case analyses (listwise deletion), where only participants that provided data on all the included variables in any given analysis were included. Sensitivity analyses were undertaken where missing data were imputed, based on the assumption that data were missing at random, using the fully conditional specification (“chained equations”).^40^ All outcome variables at all three study waves and all predictor variables were included in the imputation model. Linear regression was used to impute missing outcome data, predictive mean matching was used to impute missing data on the region; ordinal regression was used to impute missing data on age group; and logistic regression was used to impute missing data on gender. Forty imputed datasets were created. Analyses of imputed datasets provided similar results to the reported complete case analyses.

### 2.4 Ethics

Ethical approval was provided by the Newcastle University Faculty of Medical Sciences Research Ethics Committee (ref 1910/2410).

## 3 RESULTS

In this section, we will first describe the participant characteristics (Table 1), and then explore whether the data support or refute our hypotheses. Of the 477 participants who provided data at T1, 65% reported being female, 64% were under 25 and 76% reported being white. Most participants completed their FiY1 in the same region as their medical school (75%), and just under two‐thirds stayed in the same region between FiY1 and F1 (62%).

**TABLE 1:** Summary of participant characteristics at T1, T2 and T3.

Variable	Provided data at T1	Provided data at T2	Provided data at T3
	N = 477	N = 215	N = 172
Gender			
Male, n (%)	161 (33.7)	70 (32.6)	51 (29.7)
Female, n (%)	310 (65.0)	143 (66.5)	117 (68.0)
Missing, n (%)	6 (1.3)	2 (0.9)	4 (2.3)
Age group			
Under 25, n (%)	305 (63.9)	124 (57.7)	108 (62.8)
25 or over, n (%)	169 (35.4)	89 (41.4)	62 (36.0)
Missing, n (%)	3 (0.6)	2 (0.9)	2 (1.2)
Ethnic group			
White, n (%)	362 (75.9)	160 (74.4)	131 (76.2)
Asian, n (%)	73 (15.3)	33 (15.3)	25 (14.5)
Black, n (%)	11 (2.3)	7 (3.3)	7 (4.1)
Unspecified other, n (%)	2 (0.4)	1 (0.5)	1 (0.6)
Missing, n (%)	29 (6.1)	14 (6.5)	8 (4.7)
UK nation			
England, n (%)	360 (75.5)	164 (76.3)	137 (79.7)
Scotland, n (%)	65 (13.6)	23 (10.7)	15 (8.7)
Wales, n (%)	8 (1.7)	6 (2.8)	4 (2.3)
Northern Ireland, n (%)	40 (8.4)	20 (9.3)	14 (8.1)
Missing, n (%)	4 (0.8)	2 (0.9)	2 (1.2)
Completing FiY1 job in the same region as medical school			
No, n (%)	118 (24.7)	59 (27.4)	47 (27.3)
Yes, n (%)	357 (74.8)	155 (72.1)	124 (72.1)
Missing, n (%)	2 (0.4)	1 (0.5)	1 (0.6)
Staying in the same region between FiY1 and foundation training			
No, n (%)	175 (36.7)	67 (31.2)	52 (30.2)
Yes, n (%)	296 (62.0)	146 (67.9)	118 (68.6)
Missing, n (%)	6 (1.3)	2 (0.9)	2 (1.2)

Although attempts were made to access and invite all FiY1s to participate in the survey from the national FiY1 population it is not possible to provide information about non‐respondents.

### 3.1 Hypothesis 1: higher ToA is associated with better current wellbeing

There is support for Hypothesis 1: higher levels of ToA were associated with reduced levels of stress, anxiety, depression and workplace burnout. Table 2 reports the results of linear regression models fitted to examine the relationship between TAMSAD and wellbeing outcome measures (PSS, HADS‐anxiety, HADS‐depression, CBI‐work burnout and CBI‐personal burnout). The regression coefficient is the mean increase in outcome score for a one‐unit increase in the TAMSAD sum score. The R‐squared statistic (explained variation) is reported. This is the percentage of variation in the wellbeing outcomes measure that is ‘explained’ by ToA measured by the TAMSAD. We focus on the adjusted results here since the unadjusted results and adjusted results (for gender and age) are similar.

**TABLE 2:** Relationship between tolerance of ambiguity and other measured outcomes.

Outcome	Unadjusted							Adjusted for gender and age group						
	N	Coefficient	95% CI			p value	R^2^	N	Coefficient	95% CI			p value	R^2^
Perceived stress sum														
at T1	451	−0.10	−0.16	to	−0.03	0.004	1.9%	445	−0.09	−0.15	to	−0.02	0.008	1.6%
at T2	204	−0.13	−0.25	to	−0.01	0.04	2.2%	202	−0.14	−0.26	to	−0.02	0.02	2.7%
at T3	163	−0.16	−0.29	to	−0.03	0.02	3.6%	159	−0.16	−0.30	to	−0.02	0.02	3.4%
Anxiety [HADS sum]														
at T1	451	−0.07	−0.11	to	−0.02	0.003	2.0%	445	−0.06	−0.10	to	−0.01	0.009	1.6%
at T2	204	−0.08	−0.15	to	−0.01	0.03	2.2%	202	−0.08	−0.16	to	−0.01	0.03	2.4%
at T3	163	−0.08	−0.16	to	−0.003	0.04	2.5%	159	−0.07	−0.15	to	0.01	0.10	1.7%
Depression [HADS sum]														
at T1	451	−0.03	−0.06	to	−0.001	0.04	0.9%	445	−0.03	−0.07	to	−0.003	0.03	1.1%
at T2	204	0.01	−0.04	to	0.06	0.67	0.1%	202	0.01	−0.04	to	0.06	0.74	0.06%
at T3	163	−0.02	−0.08	to	0.04	0.43	0.4%	159	−0.02	−0.08	to	0.05	0.61	0.2%
CBI‐work burnout														
at T1	451	−0.39	−0.57	to	−0.21	<0.001	3.8%	445	−0.40	−0.58	to	−0.21	<0.001	3.9%
at T2	204	−0.28	−0.61	to	0.05	0.09	1.4%	202	−0.32	−0.66	to	0.01	0.05	1.9%
at T3	163	−0.43	−0.79	to	−0.08	0.02	3.5%	159	−0.50	−0.86	to	−0.14	0.007	4.6%
CBI‐personal burnout														
at T1	451	−0.18	−0.37	to	0.01	0.06	0.8%	445	−0.17	−0.36	to	0.02	0.08	0.7%
at T2	204	−0.19	−0.49	to	0.12	0.22	0.7%	202	−0.17	−0.48	to	0.14	0.27	0.6%
at T3	163	−0.34	−0.70	to	0.02	0.06	2.1%	159	−0.34	−0.70	to	0.02	0.06	2.3%

*Linear regression models fitted to examine the relationship between ToA (TAMSAD) and wellbeing (PSS, HADS‐anxiety, HADS‐depression, CBI‐work burnout and CBI‐personal burnout) at T1, T2 and T3. Adjusted (for gender and age) and unadjusted results reported. R‐squared statistic (explained variation) reported*.

Interpretation: Regression coefficient indicates the change in the outcome that is associated with a 1‐point increase in ToA (measured using the TAMSAD scale).

Higher TAMSAD scores at T1 were associated with lower PSS sum scores (−0.09, R2 = 1.6%, p = 0.008). This means that a one‐point rise in TAMSAD score is associated with a 0.09‐point reduction in the PSS sum score at T1. Higher TAMSAD scores at T1 were also associated with lower HADS‐anxiety sum scores (−0.06, R2 = 1.6%, p = 0.009), lower HADS‐depression sum scores (−0.03, R2 = 1.1% p = 0.03) and lower CBI‐work burnout sum scores (−0.40, R2 = 3.9% p < 0.001) at T1. For each of these relationships, the reported R‐squared values are less than 9% and are therefore considered to be small.^41^

### 3.2 Hypothesis 2: higher ToA is associated with better future wellbeing

There is support for Hypothesis 2: higher levels of ToA at T1 were associated with lower levels of stress and anxiety at T2 and, for stress, this relationship continued to persist at T3. There was also evidence of an association between higher levels of ToA at T1 and lower levels of work‐related burnout at T3. Higher TAMSAD scores at T1 were associated with lower PSS sum scores at T2 (−0.14, R2 = 2.7%, p = 0.02) and at T3 (−0.16, R2 = 3.4%, p = 0.02), and lower HADS‐anxiety sum scores at T2 (−0.08, R2 = 2.4%, p = 0.03). However, this association did not persist into T3 (−0.07, R2 = 1.7%, p = 0.10). The reported R‐squared values for these relationships are small.^41^

There was no evidence of an association between TAMSAD scores at T1 and HADS‐depression sum scores at T2 (0.01, R2 = 0.06%, p = 0.74), or T3 (−0.02, R2 = 0.2%, p = 0.61). There was evidence of an association between higher TAMSAD scores at T1 and lower CBI‐work burnout sum scores at T3 (−0.50, R2 = 4.6%, p = 0.007). Higher TAMSAD scores at T1 were not associated with a lower CBI‐personal sum scores at T2 (−0.17, R2 = 0.6%, p = 0.27), or lower CBI‐personal sum scores at T3 (−0.34, R2 = 2.3%, p = 0.06).

### 3.3 Hypothesis 3: individual factors (age and gender) moderate the relationship between ToA and wellbeing

There is support for Hypothesis 3: that individual factors such as age and gender moderate the relationship between ToA and wellbeing. We acknowledge the complicated nature of these analyses and provide a succinct summary (Table 3) which shows all 60 potential relationships evaluated as part of this exploratory analysis. There was no significant evidence of a difference across age groups for the relationship between TAMSAD and either PSS or HADS anxiety at T1. However, at T2, higher TAMSAD scores were associated with lower PSS scores (−0.28, 95% CI: −0.46 to −0.10) for those over 25, but not for those under 25 (−0.02, 95% CI: −0.18 to 0.13), and also for lower HADS‐anxiety scores (−0.19, 95% CI: −0.30 to −0.08) for those over 25, but not for those under 25 (0.001, 95% CI: −0.10 to 0.10). Similarly, at T3, higher TAMSAD scores were associated with lower PSS scores (−0.38, 95% CI: −0.62 to −0.14), for those over 25, but not for those under 25 (−0.03, 95% CI: −0.18 to 0.13), and also for lower HADS‐anxiety scores (−0.17, 95% CI: −0.29 to −0.05) for those over 25, but not for those under 25 (−0.003, 95% CI: −0.10 to 0.09).

**TABLE 3:** Moderation analyses demonstrating impact of gender, age, relocation between medical school and FiY1, and relocation between FiY1 and F1 on the relationship between ToA and wellbeing (stress, anxiety, depression and burnout).

Outcome	Potential moderator			
	Gender	Age	Same region medical school & FiY1	Same region FiY1 & F1
Perceived stress sum				
at T1 [N = 442 to 445]	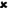	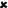	NoRel for “No” 0.03 (−0.10 to 0.16) YesRel for “Yes” −0.14 (−0.21 to −0.06)	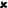
at T2 [N = 202]	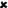	NoRel <25 −0.02 (−0.18 to 0.13) YesRel >25 −0.28 (−0.46 to −0.10)	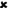	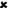
at T3 [N = 159]	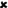	‐ NoRel <25 −0.03 (−0.18 to 0.13) YesRel >25 −0.38 (−0.62 to −0.14)	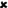	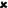
Anxiety [HADS sum]				
at T1 [N = 442 to 445]	NoRel men 0.004 (−0.05 to 0.06) YesRel women −0.11 (−0.17 to −0.05)	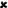	NoRel for “No” 0.02 (−0.07 to 0.11) YesRelfor “Yes” −0.09 (−0.13 to −0.04)	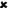
at T2 [N = 202]	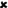	NoRel <25 0.001 (−0.10 to 0.10) YesRel >25 −0.19 (−0.30 to −0.08)	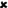	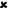
at T3 [N = 159]	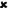	NoRel <25 −0.003 (−0.10 to 0.09) YesRel >25 −0.17 (−0.29 to −0.05)	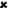	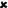
Depression [HADS sum]				
at T1 [N = 442 to 445]	NoRel men 0.007 (−0.04 to 0.05) YesRel women −0.07 (−0.11 to −0.03)	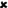	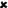	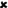
at T2 [N = 202]	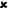	YesRel <25 0.09 (0.02 to 0.15) YesRel >25 −0.08 (−0.16 to −0.01)	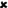	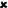
at T3 [N = 159]	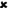	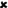	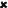	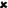
CBI‐work burnout				
at T1 [N = 442 to 445]	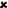	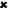	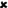	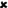
at T2 [N = 202]	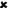	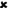	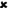	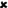
at T3 [N = 159]	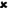	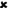	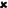	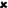
CBI‐personal burnout				
at T1 [N = 442 to 445]	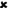	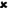	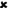	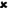
at T2 [N = 202]	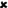	NoRel <25 0.19 (−0.21 to 0.58) YesRel >25 −0.64 (−1.11 to −0.16)	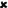	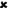
at T3 [N = 159]	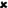	NoRel <25 0.04 (−0.39 to 0.46) YesRel >25 −0.85 (−1.48 to −0.23)	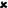	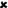

*For each outcome (rows) the table indicates whether there is statistically significant evidence of moderation (potential moderators in columns). Where there is moderation the relationship between the TAMSAD score and the measure of wellbeing is reported separately for each sub‐group of the potential moderator using the regression coefficient and 95% confidence interval. Separate p‐values were not reported for each subgroup in order to avoid multiple testing. No statistically significant evidence of moderation (*i.e.*, p‐value for the test of interaction is > 0.05) is indicated by a cross (* 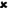 *) in the appropriate cell*.

NoRel = no relationship, YesRel = evidence of a relationship.

In female participants, but not males, there was a relationship between higher ToA and lower levels of anxiety and depression at T1. Higher TAMSAD scores were associated with lower HADS‐anxiety scores (−0.11, 95% CI: −0.17 to −0.05) and HADS‐depression scores (−0.07, 95% CI: −0.11 to −0.03) for females, but not HADS‐anxiety (0.004, 95% CI: −0.05 to 0.06) or HADS‐depression scores (0.007, 95% CI: −0.04 to 0.05) for males. There was no evidence of a difference in this relationship based on gender at T2 or T3.

### 3.4 Hypothesis 4: workplace factors (recent change in working environment) moderates the relationship between ToA and wellbeing

There is partial evidence to support Hypothesis 4. For individuals completing FiY1 in the same region as their medical school, there was a relationship between higher levels of ToA and lower levels of stress and anxiety during T1. However, this relationship was not present for those moving between medical school and FiY1.

There was evidence of a difference based on whether participants completed their FiY1 post in the same region as their medical school for the relationship both between TAMSAD and PSS and between TAMSAD and HADS‐anxiety at T1. Higher TAMSAD scores were associated with lower PSS scores (−0.14, 95% CI: −0.21 to −0.06) and lower HADS anxiety scores (−0.09, 95% CI: −0.13 to −0.04) for those completing FiY1 in the same region as their medical school, but not if participants moved between medical school and FiY1 PSS: (0.03, 95% CI: −0.10 to 0.16); HADS‐anxiety: (0.02, 95% CI: −0.07 to 0.11). There was no evidence of difference in this relationship for this potential moderator at T2 or T3.

## 4 DISCUSSION

This study aimed to explore the cross‐sectional and longitudinal relationship between ToA and wellbeing in newly qualified doctors, and the individual or workplace factors that moderate this relationship in the context of the COVID‐19 pandemic.

Higher levels of ToA were associated with lower levels of stress, anxiety, depression and workplace burnout in newly qualified doctors. This confirms the findings of a previous systematic review^7^ however, our study utilises robust methodology, with valid measurement scales and a longitudinal follow‐up period. This study demonstrates for the first time that higher levels of ToA in newly qualified doctors at T1 were associated with lower levels of stress, anxiety and work‐related burnout at later measurement points (T2 and T3). The small R squared values^41^ likely reflect a complex relationship between ToA and wellbeing, with multiple factors besides ToA influencing stress, anxiety, depression or burnout. Given the context in which this study was conducted, the exploratory nature of the moderator analyses and the number of tests undertaken, evidence of moderation should be treated as provisional, requiring replication in future studies in order to have credence.

There is growing evidence of moderators to the relationship between ToA and wellbeing. Although some have been suggested previously, such as resilience,^12,13,14^ exploratory analyses in this paper have identified variables as potential moderators for the first time. Being older and female appears to make it more likely that reduced ToA will be associated with reduced wellbeing in this population. The impacts of workplace moderators remain unclear. Our study suggested that staying in the same region between medical school and FiY1 made it more likely that reduced ToA will be associated with reduced wellbeing.

It is difficult to quantify the impact of the COVID‐19 pandemic on these results and this will require testing through future research. The rapid and novel development of FiY1 roles are unlikely to be repeated and this was an unparalleled opportunity to conduct this study in this population via the ‘natural experiment’ provided by COVID‐19.^42^

Much has been published about the COVID‐19 pandemic, and its impact on medical professionals. In the UK it has been reported that over the first year of the pandemic levels of burnout, overwork, distress and trauma significantly increased in doctors.^43^ Subsequently it may be that this context influenced all of the relationships explored in this study. The results should be seen as exploratory and interpreted with this in mind. Despite this, we argue that our findings provide novel insights that will require further testing through future research.

### 4.1 Development of conceptual model

**FIGURE 2:**
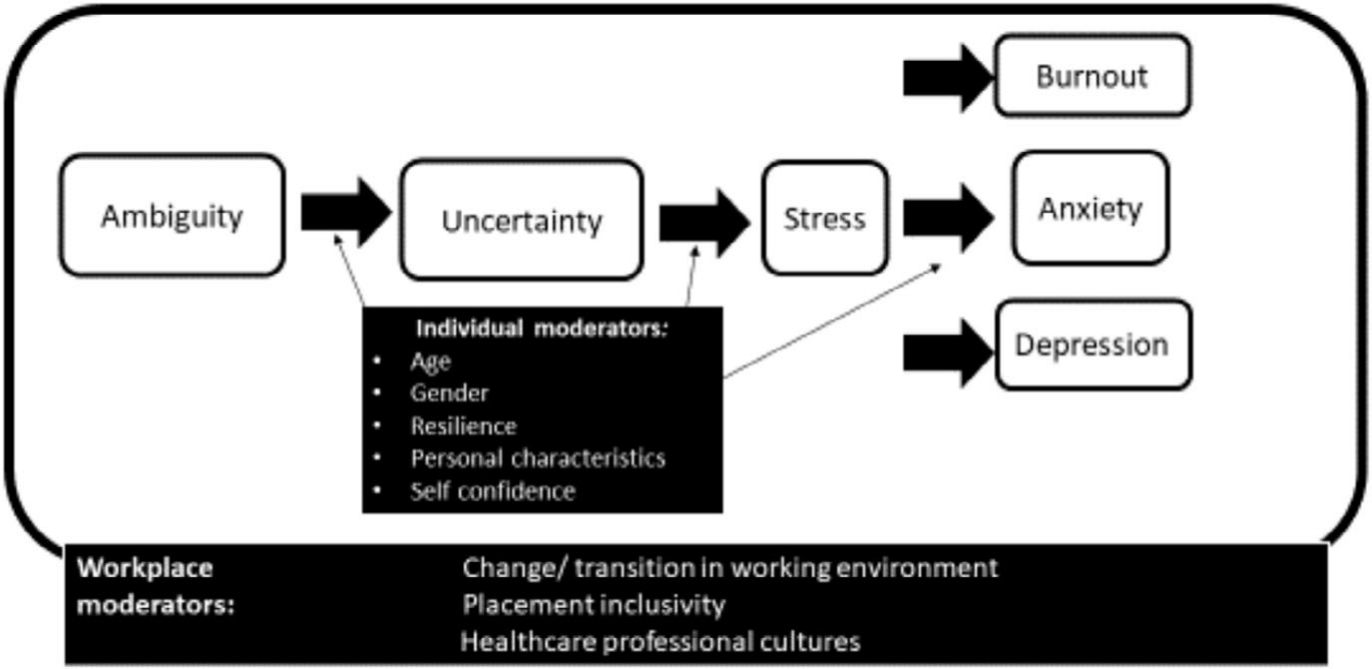
Conceptual model describing the relationship between ToA and wellbeing including potential moderators.

Carefully conceptualised and rigorously designed research programmes are needed to progress our understanding of the relationship between ToA and wellbeing.^7^, ^44^ We propose an updated conceptual model to describe the relationship between ToA and wellbeing, based on the empirical findings from this study, the wider literature and our a priori knowledge as researchers and clinicians (see Figure 2). This builds on a previously published conceptual model.^7^

This study provides evidence of a longitudinal relationship between ToA and wellbeing, in particular stress and anxiety, which is reflected in this updated conceptual model. Ambiguity is a source of uncertainty, and reduced ToA can progress to stress and anxiety. The arrows in this model reflect this. There is also likely to be a relationship between ToA and both burnout and depression.

There are likely to be individual and workplace factors that moderate the relationship between ToA and wellbeing,^12^, ^13^, ^17^, ^18^, ^19^, ^22^ as suggested previously. Although this study provides some evidence that individual factors such as gender and age may moderate the relationship, further clarification of moderators within this conceptual model is required. Furthermore, we reiterate that given the number of statistical tests undertaken, the moderation analyses are exploratory in nature and the findings need to be replicated in other studies to have credence.

### 4.2 Strengths and limitations

The strength of this study is the longitudinal study design, which uniquely examines the relationship between ToA and a range of wellbeing measures and moderators of the relationship. We identified three main study limitations. Firstly the sample sizes in the later time points (T2 and T3) were much reduced with only those completing earlier questionnaires included in the subsequent analysis. However, the demographic details of participants at each time point reveal broadly similar participant characteristics. Secondly, the follow‐up period was also only four months, limiting our ability to make interpretations about any longer‐term relationship. However, the time period represents a significant transition for this population which may be a focus for future interventions. Finally, the individuals completing these surveys were being rapidly graduated to novel and new roles (which were themselves unclear and uncertain) at a time of a global pandemic. Despite an evaluation of the FiY1 programme indicating that on starting F1 there was no difference in wellbeing measures for those completing or not completing FiY1,^27^ it is possible that individuals completing these novel roles were experiencing a significant degree of stress. Thus, while conducting this study at this time provides an unprecedented opportunity to examine individual responses to ambiguity and uncertainty, it will be important to replicate these findings outside of the global pandemic context.

## 5 CONCLUSIONS

The findings that being older and staying in the same region made it more likely that reduced ToA would progress to reduced wellbeing were contrary to our initial hypothesis. This may challenge the assumption that ‘local graduates’ will adapt to their clinical workplace without significant detriment to their wellbeing. We have used these findings to refine a conceptual model describing the relationship between ToA and wellbeing, which can inform future research and the development of educational interventions to support doctors to develop more positive ToA and improve their wellbeing. Further work is needed to clarify the impact of workplace factors on the relationship between ToA and wellbeing, and how rotational training schemes and/or workplaces could be designed differently to better support trainees to manage the ambiguity inherent within medical practice.

## AUTHOR CONTRIBUTIONS

**Jason Hancock:** Conceptualization; writing—original draft; methodology; validation; writing—review and editing; formal analysis; project administration. **Obioha Ukoumunne C:** Methodology; validation; formal analysis; writing—review and editing; supervision. **Bryan Burford:** Conceptualization; investigation; funding acquisition; writing—review and editing; methodology; formal analysis; resources. **Gillian Vance:** Funding acquisition; investigation; conceptualization; writing—review and editing; resources. **Thomas Gale:** Funding acquisition; investigation; conceptualization; writing—review and editing. **Karen Mattick:** Conceptualization; investigation; funding acquisition; writing—review and editing; validation; methodology; supervision; resources.

## CONFLICT OF INTEREST STATEMENT

None of the authors has a conflict of interest to disclose.

## Supporting information


**Supplementary table 1:** Validity argument for the use of scales in the FiY1 population.
**Supplementary table 2:** Questionnaire scoring and interpretation.

## Data Availability

The datasets generated and/or analysed during the current study are available in the following repository: https://data.ncl.ac.uk/articles/dataset/Questionnaire_data_files_for_study_of_interim_Foundation_Year_1_FiY1_doctors_transition_to_practice_in_2000/22537099.
