# Supplementary material for: Tolerance of ambiguity and psychological wellbeing in newly qualified doctors: An analysis over multiple time points
Source: Med Educ. 2025 Jun 17;59(10):1094–104. doi: 10.1111/medu.15743 (PMC12438002; doi:10.1111/medu.15743)
Supplement: Supplementary file 1 — Supplementary table 1: Validity argument for the use of scales in the FiY1 population. Supplementary table 2: Questionnaire scoring and interpretation. [file MEDU-59-1094-s001.docx]

# Appendices

## Supplementary table 1: Validity argument for the use of scales in the FiY1 population

The validity of these scales have been evaluated utilising a framework by the American Educational Research Association, American Psychological Association, National Council on Measurement in Education (1999) applied to the medical education context by Downing (2003).

*Downing, S. (2003). Validity: On the meaningful interpretation of assessment data. Medical Education, 37, 830–837.*

TAMSAD

| Form of validity evidence | Rationale |
| --- | --- |
| Content related | Items derived from an analysis of the education literature, from medical education theory and from existing tolerance of ambiguity scales.  Pilot work involved the input of academic staff working in medical education and from medical practitioners working in hospital and community settings. |
| Response process | Considered during pilot work and through discussion with academic staff.  Also considered prior to psychometric scale study when ten early career doctors were asked to read items and comment on those that were difficult to understand or answer. This resulted in removal of several items and rewording of others. |
| Internal structure of  the scale | Scale was found to be acting unidimensionally in the original psychometric scale development study (referenced below). In this study the Cronbach’s alpha = 0.80. |
| Relationship to other variables | The psychometric scale development study demonstrated that TAMSAD scores were higher in foundation year 2 doctors surveyed compared to first, third and fourth year medical students. While it is debated if ToA or the related construct of UT increases during medical training this has been demonstrated in a number of studies. |
| Consequences of using the assessment  scale | Minimal. Takes between 5-10 minutes to complete 29 item scale |

*Hancock, J., Roberts, M., Monrouxe, L. and Mattick, K. (2015) Medical student and junior doctors’ tolerance of ambiguity: development of a new scale, Advances in Health Sciences Education, 20(1): 113‐130.*

PSS

| Form of validity evidence | Rationale |
| --- | --- |
| Content related | The initial perceived Stress Scale (PSS) contained 14 items and was developed by the authors in 1983 as a measure of the degree to which situations in one's life are appraised as stressful. This 14 item scale was subsequently shortened to a 10 item version using factor analysis which is utilised in this study. |
| Response process | Considered during pilot work, through discussion with FiY1 doctors. Asked to read and complete all scale items and comment on those that were difficult to understand or answer. |
| Internal structure of  the scale | PSS-10 item scale has a reported internal consistency/ Cronbach’s alpha of 0.78 |
| Relationship to other variables | Higher PSS scores have been correlated with life event scores, depressive and anxiety symptomatology, and utilisation of health services. When compared to a depressive symptomatology scale, the PSS was found to measure a different and independent predictive construct. |
| Consequences of using the assessment  scale | Minimal. Takes between 5-10 minutes to complete. |

*Cohen S, Kamarck T Mermelstein R. A global measure of perceived stress. Journal of Health and Social Behavior, 1983;24: 385-396.*

*Cohen, S. and Williamson, G. Perceived Stress in a Probability Sample of the United States. Spacapan, S. and Oskamp, S. (Eds.) The Social Psychology of Health. Newbury Park, CA: Sage, 1988.*

*Tao Y, Cheng Z, Wang C, Liu T, Yan M, Huang X, Jian S, Sun L and Chen Z. Perceived stress and psychological disorders in healthcare professionals: a multiple chain mediating model of effort-reward imbalance and resilience. Front. Public Health. 11:1320411. doi: 10.3389/fpubh.2023.1320411*

HADS anxiety and depression

| Form of validity evidence | Rationale |
| --- | --- |
| Content related | Seven statements written to capture the experience of anxiety as it may manifest in a medical setting whilst avoiding reliance on purely somatic symptoms.  Seven statements written to capture the experience of depression as it may manifest in a medical setting whilst avoiding reliance on purely somatic symptoms. |
| Response process | Considered during pilot work, through discussion with FiY1 doctors. Asked to read and complete all scale items and comment on those that were difficult to understand or answer. |
| Internal structure of  the scale | Cronbach’s alpha of anxiety subscale 0.80 demonstrated in a large population (92,100) participants.  Cronbach’s alpha of depression subscale 0.76 demonstrated in a large population (92,100) participants. |
| Relationship to other variables | In one study by Marfell (2019) Medical students were asked to complete the full HADS and also interviewed using the Schedules for Clinical Assessment in Neuropsychiatry (SCAN). This study concluded that HADS is an appropriate tool for assessment of depression and anxiety in the similar medical student (UK) cohort. |
| Consequences of using the assessment  scale | Minimal. Takes between 5-10 minutes to complete. |

*Marfell NR. Measuring depression and anxiety in medical students: Is HADS an appropriate tool? MPhil Thesis, Cardiff University, 2019*

*Mykletun, A., Stordal, E. and Dahl A. Hospital Anxiety and Depression (HAD) scale: factor structure, item analyses and internal consistency in a large population. Br J Psychiatry. 2001 Dec: 179:540-4. doi: 10.1192/bjp.179.6.540.*

*Stern A. (2014). The Hospital Anxiety and Depression Scale. Occupational Medicine, Volume 64, Issue 5, July 2014, Pages 393–394, https://doi.org/10.1093/occmed/kqu024*

Copenhagen Burnout Inventory, CBI – personal and work related

| Form of validity evidence | Rationale |
| --- | --- |
| Content related | Items within the CBI were developed by the authors following a review of the existing theory, the literature and following a review of existing measures of burnout (such as the Maslach Burnout Inventory). Items related to personal burnout were formulated to ensure they could be answered universally, items related to work related burnout were formulated assuming that the respondents would be engaging in paid work of some form. The initial CBI study included healthcare staff working in both a ‘somatic’ and a psychiatric hospital. |
| Response process | Considered during pilot work, through discussion with FiY1 doctors. Asked to read and complete all scale items and comment on those that were difficult to understand or answer. |
| Internal structure of  the scale | Within the initial CBI study the internal consistency of these subscales were:  Personal burnout Cronbach’s alpha: 0.87  Work related burnout Cronbach’s alpha: 0.87 |
| Relationship to other variables | It has been shown that there is a significant association between burnout measured with the CBI and high efforts and over commitment |
| Consequences of using the assessment  scale | Minimal. Takes between 5-10 minutes to complete. |

*Kristensen TS, Borritz M, Villadsen E, Christensen KB. Copenhagen Burnout Inventory: a new tool for the assessment of burnout. Work Stress. 2005;19:192–207.*

*Odagiri, Y., Shimomitsu, T., Ohya, Y., & Kristensen, T. S. Overcommitment and high effort are strongly associated with burnout among Japanese nurses. International Journal of Behavioral Medicine. 2004. 11, Suppl., p. 214.*

## Supplementary table 2: Questionnaire scoring and interpretation

| Scale | Total sum | Scoring | Time frame examined |
| --- | --- | --- | --- |
| Perceived Stress Scale (PSS) | 40 | Low stress: 0 – 13  Moderate stress: 14 – 26  High stress: 27 - 40 [Cohen et al, 1983 & 1988] | Self reported experiences (stress) over the last month. |
| HADS – anxiety | 21 | Different categorical cut offs can be used.  Non cases: ≤ 7  Mild: 8-10  Moderate: 11-14  Severe: 15-21 [Stern, 2014]  In a population of medical students  Anxiety indicated by a score ≥13 [Marfell, 2019] | Self reported symptoms over the last week. |
| HADS – depression | 21 | Different categorical cut offs can be used.  Non cases: ≤ 7  Mild: 8-10  Moderate: 11-14  Severe: 15-21 [Stern, 2014]  In a population of medical students  Depression indicated by a score ≥7 [Marfell, 2019] | Self reported symptoms over the last week. |
| Copenhagen Burnout Inventory  CBI – personal | 100 | Moderate: 50 to 74  High: 75–99  Severe burnout: 100 [Kristensen et al, 2005] | No specified time frame, but questions ask *“How often do you feel…”* |
| Copenhagen Burnout Inventory  CBI – work-related | 100 | Moderate: 50 to 74  High: 75–99  Severe burnout: 100 [Kristensen et al, 2005] | No specified time frame, but questions ask *“How often do you feel…”* |
| TAMSAD | 100 | No published ‘cut offs’ or categorical data.  A 5 point mean difference in TAMSAD scores observed between first year medical students and F2 doctors [Hancock et al, 2015]. | Single point in time |

*Cohen S, Kamarck T Mermelstein R. A global measure of perceived stress. Journal of Health and Social Behavior, 1983;24: 385-396.*

*Cohen, S. and Williamson, G. Perceived Stress in a Probability Sample of the United States. Spacapan, S. and Oskamp, S. (Eds.) The Social Psychology of Health. Newbury Park, CA: Sage, 1988.*

*Hancock, J., Roberts, M., Monrouxe, L. and Mattick, K. (2015) Medical student and junior doctors’ tolerance of ambiguity: development of a new scale, Advances in Health Sciences Education, 20(1): 113‐130.*

*Kristensen TS, Borritz M, Villadsen E, Christensen KB. Copenhagen Burnout Inventory: a new tool for the assessment of burnout. Work Stress. 2005;19:192–207.*

*Marfell NR. Measuring depression and anxiety in medical students: Is HADS an appropriate tool? MPhil Thesis, Cardiff University, 2019*

## *Stern A. (2014). The Hospital Anxiety and Depression Scale. Occupational Medicine, Volume 64, Issue 5, July 2014, Pages 393–394,* [*https://doi.org/10.1093/occmed/kqu024*](https://doi.org/10.1093/occmed/kqu024)
